# Vesicular Stomatitis Virus Enters Cells through Vesicles Incompletely Coated with Clathrin That Depend upon Actin for Internalization

**DOI:** 10.1371/journal.ppat.1000394

**Published:** 2009-04-24

**Authors:** David K. Cureton, Ramiro H. Massol, Saveez Saffarian, Tomas L. Kirchhausen, Sean P. J. Whelan

**Affiliations:** 1 Departments of Microbiology and Molecular Genetics, Harvard Medical School, Boston, Massachusetts, United States of America; 2 Program in Virology, Harvard Medical School, Boston, Massachusetts, United States of America; 3 Children’s Hospital, Boston, Massachusetts, United States of America; 4 Department of Cell Biology, Harvard Medical School, Boston, Massachusetts, United States of America; 5 Immune Disease Institute, Harvard Medical School, Boston, Massachusetts, United States of America; The Salk Institute for Biological Studies, United States of America

## Abstract

Many viruses that enter cells by clathrin-dependent endocytosis are significantly larger than the dimensions of a typical clathrin-coated vesicle. The mechanisms by which viruses co-opt the clathrin machinery for efficient internalization remain uncertain. Here we examined how clathrin-coated vesicles accommodate vesicular stomatitis virus (VSV) during its entry into cells. Using high-resolution imaging of the internalization of single viral particles into cells expressing fluorescent clathrin and adaptor molecules, we show that VSV enters cells through partially clathrin-coated vesicles. We found that on average, virus-containing vesicles contain more clathrin and clathrin adaptor molecules than conventional vesicles, but this increase is insufficient to permit full coating of the vesicle. We further show that virus-containing vesicles depend upon the actin machinery for their internalization. Specifically, we found that components of the actin machinery are recruited to virus-containing vesicles, and chemical inhibition of actin polymerization trapped viral particles in vesicles at the plasma membrane. By analysis of multiple independent virus internalization events, we show that VSV induces the nucleation of clathrin for its uptake, rather than depending upon random capture by formation of a clathrin-coated pit. This work provides new mechanistic insights into the process of virus internalization as well as uptake of unconventional cargo by the clathrin-dependent endocytic machinery.

## Author Summary

Clathrin-dependent endocytosis accounts for the majority of uptake from the plasma membrane. However, many viruses that infect cells through an endocytic route are larger than the dimensions of a typical clathrin-coated vesicle. Working with vesicular stomatitis virus, we determined how this cargo enters cells. We present evidence that VSV induces its own uptake by the clathrin-dependent endocytic machinery following binding to the plasma membrane. The clathrin-coated vesicles that contain virus differ from vesicles that internalize conventional clathrin dependent cargo such as LDL and transferrin. Specifically, we show that VSV particles are internalized by vesicles that are only partially coated with clathrin, rather than the complete coat found on conventional vesicles. We show that the clathrin-dependent endocytic adaptor AP-2 is required for entry. Finally, we show for the first time that actin is recruited to virus-containing pits and that particle internalization depends upon actin function. Our work provides new mechanistic insights into VSV entry that may be directly relevant in understanding the clathrin dependent uptake of other viruses.

## Introduction

Clathrin-mediated endocytosis is the major transport pathway from the plasma membrane to early endosomes. In this process, the plasma membrane typically invaginates into a clathrin-coated pit, in which adaptor molecules bridge the interaction of clathrin with cargo. During invagination, the ubiquitous adaptor protein complex, AP-2, binds to specific sorting signals in the cytosolic tails of membrane proteins, and to phospholipids and clathrin. As the coated pit grows, additional clathrin and adaptor molecules assemble to form an enclosed and fully coated structure [1]. Separation of this pit from the plasma membrane requires a large GTPase, dynamin [2],[3]; clathrin rapidly uncoats from the resulting coated vesicle through action of the Hsc70 ATPase and its cofactor, auxilin [4],[5].

Infection by many viruses is sensitive to inhibition of the clathrin pathway. Among the best-studied examples is vesicular stomatitis virus (VSV), a prototype of the *Rhabdoviridae*. Initial evidence for the clathrin-dependent uptake of VSV comes from electron micrographs that show viral particles present within coated pit structures [6],[7]. More recent experiments show that viral gene expression is inhibited by a dominant negative mutant of epidermal growth factor receptor pathway substrate clone 15 (Eps15), a protein involved in clathrin-mediated endocytosis, or by treatment of cells with siRNA targeted to clathrin heavy chain [8]. A functional clathrin pathway is therefore required for efficient viral infection.

Coated pit assembly *in vivo* has been followed in detail by live cell imaging, using individual, fluorescently labeled low density lipoprotein (LDL) and reovirus particles to visualize cargo [1]. The kinetics of internalization are in both cases consistent with capture of the cargo by randomly initiating coated pits. Moreover, the amount of clathrin required to complete coated pit assembly scales as the area needed to engulf particles having the relative diameters of the two ligands. In studies using the same cell line, coated pits containing influenza A particles were reported to resemble those lacking virions (and presumed to contain other cargo molecules), but internalization of the virus appeared to occur through pits that formed directly at the site of virus binding, as if the influenza virus induced its own uptake [9]. The differences in uptake kinetics between LDL or reovirus and influenza virus suggest that there may be multiple modes of coated-pit initiation and that distinct initiation modes may entrain distinct assembly mechanisms and possibly distinct destinations for the endocytosed cargo.

Influenza and reovirus particles are both roughly spherical and not greatly different in size (120 and 85 nm diameter, respectively), so particle dimensions alone probably cannot explain the apparently different uptake modalities. VSV is a bullet-shaped particle, 180×70 nm, much longer than the diameter of influenza or reovirus and comparable in cross section. To probe the limits and correlates of alternative endocytic mechanisms, we have determined the kinetics of VSV endocytosis into clathrin-coated vesicles. Vesicles internalizing VSV appear to contain insufficient clathrin to coat fully a virus-containing vesicle. The coated pits recruit actin and associated proteins in a step that is essential for release of the vesicles from the plasma membrane. We integrate these observations, together with evidence from electron microscopy, into a model of VSV internalization. We conclude that VSV and potentially other cargo are internalized through an altered mode of clathrin-based endocytosis.

## Results

### Live cell imaging of VSV entry

**Figure 1:**
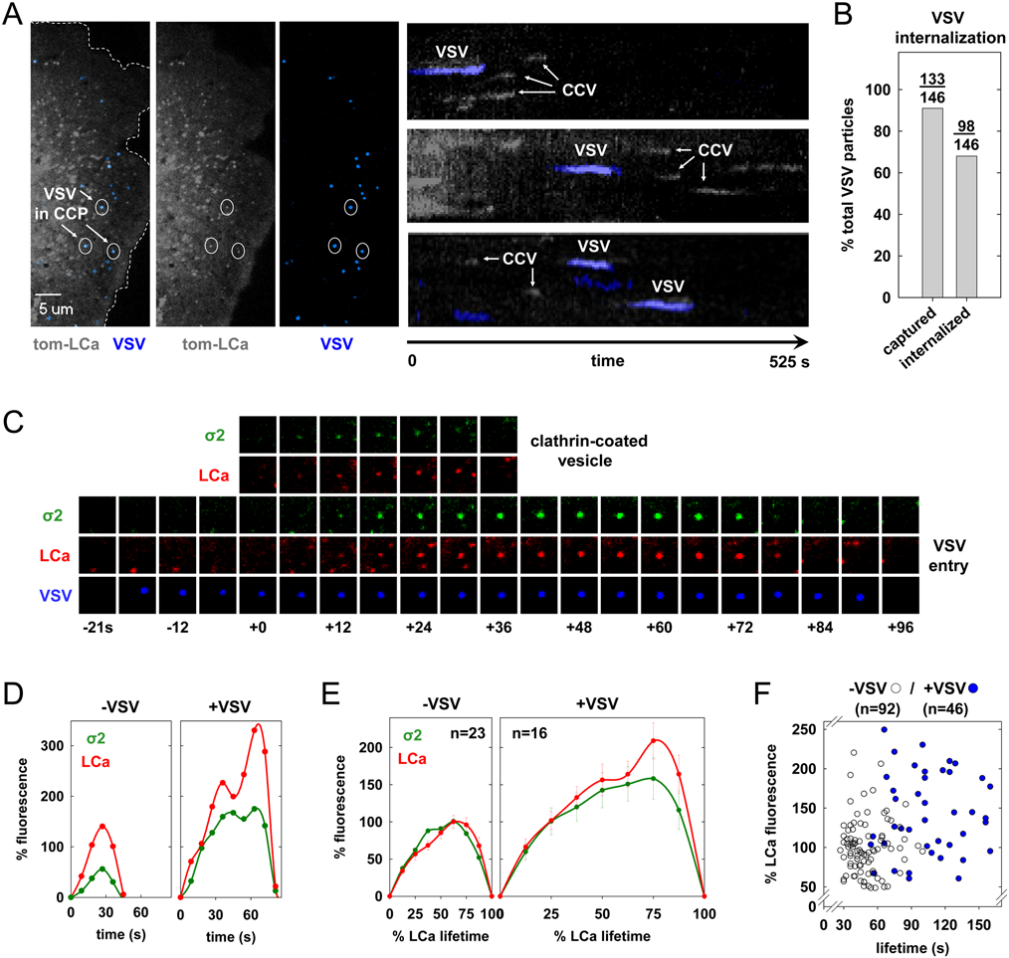
Live cell imaging of clathrin-dependent endocytosis of single VSV particles. (A) Split channel images of a single BSC-1 cell (left and also in Video S1) transiently expressing tom-LCa (grey) highlight 3 virus particles (blue, circled) during internalization by clathrin-coated pits (CCPs). Kymographs (right) of sections of the cell surface showing tom-LCa fluorescence over time for clathrin-coated vesicles (CCV) containing and lacking virus. (B) A graph of the % of virus particles captured by a CCP or internalized by a CCV was plotted for 146 particles that attached to 28 different cells during image acquisition. (C) A tile view of images of a BSC-1 cell co-expressing σ2-eGFP (green) and tom-LCa (red) cropped from a time-lapse movie (Video S2), showing VSV appearing at the cell surface (time, t = −18 s) relative to the point (t = 0) of clathrin detection above background. (D) A graph of the kinetics of AP-2 and clathrin recruitment to the CCPs of panel C. Fluorescence intensities were plotted relative to the time of clathrin detection, and are expressed as a % of the average maximum clathrin observed in all pits lacking virus. The points are a weighted average of fluorescence intensities calculated as described in Materials and Methods. (E) A graph of the average kinetics of LCa and σ2 recruitment to CCPs containing (right, from 4 cells) or lacking (left, from 2 of the 4 cells) virus. Average fluorescence intensity and time are expressed as a % relative to CCV lacking virus observed in the same cells. Fluorescence intensity was calculated at 8 equally-spaced intervals and is plotted+/−standard error. (F) A graph of the clathrin fluorescence relative to vesicle lifetime for CCPs lacking (open circles) or containing (blue) virus. Fluorescence was expressed as a % relative to the average maximum for tom-LCa in pits lacking VSV. The average lifetime for pits containing virus was 110+/−44 s (15 cells), which was statistically distinct (Student's t-test: p = 2e-12) to that for pits lacking virus (51+/−16 s from 8 of the same cells). The peak clathrin fluorescence intensities in pits containing and lacking virus was 155+/−69 and 100+/−34, respectively. The difference between these values is statistically significant (Student's t-test: p = 1e-6).

To image the internalization of single VSV particles in live cells, we conjugated the fluorescent dye, Alexa Fluor 647 to purified virions. This labeling process did not significantly reduce viral titer as measured by plaque assay (Figure S1A). Examination of labeled VSV particles by spinning disc confocal fluorescent microscopy showed distinct, diffraction-limited puncta with a single Gaussian distribution of fluorescence intensities (Figure S1B). This result is consistent with a lack of aggregates and the presence of single virus particles. To examine how clathrin-coated pits internalize VSV, we infected BSC-1 cells stably expressing tomato-clathrin light chain A1 (tom-LCa) and acquired images from the bottom surface of cells using a spinning disc confocal microscope. Single VSV particles attached to cells, and over the 6–10 min. time course of 28 time-lapse videos, more than 90% (133/146) of the attached particles associated with tom-LCa (Figure 1A, B, S3). Moreover, the tom-LCa fluorescence signal increased over time and then abruptly disappeared as the vesicle uncoated (Figure 1A, C, Videos S1, S2). More than 70% (98/133) of the captured particles underwent rapid, directed movement shortly after disappearance of the clathrin signal, indicating intracellular transport of virus-containing vesicles (Videos S1, S2). The few particles that failed to associate with clathrin remained at the cell surface and did not exhibit this rapid, directed motion. Thus, clathrin-mediated endocytosis accounts for the uptake of most attached VSV particles.

### AP-2 is a functional adaptor for productive internalization of VSV through clathrin-coated pits

To determine if AP-2 is present in clathrin-coated pits that contain VSV, we imaged virus internalization in cells expressing tom-LCa and the σ2 subunit of AP-2 fused to eGFP (σ2-eGFP). Without exception, σ2-eGFP appeared and disappeared along with tom-LCa (Figure 1C–E), demonstrating that VSV internalization occurs in clathrin-coated pits that contain AP-2. We examined the abundance and the association kinetics of tom-LCa and σ2-eGFP in pits that contain virus and compared them with pits that lack virus. Clathrin and AP-2 recruitment are initially similar, independent of virus (Figure 1D, E), but virus-containing pits often show a transient plateau in the accumulation of tom-LCa followed by a subsequent burst of clathrin accumulation (Figure 1D, E). Pits containing virus showed longer lifetimes and on average contained more clathrin and AP-2 than pits in the same cell that lacked virus (Figure 1E, F). Combined with other experiments in cells expressing tom-LCa (see below), we measured clathrin accumulation and pit lifetime for 46 pits containing virus and compared them with 92 that lacked virus. Pits containing virus recruit on average 1.5-fold more clathrin, and have lifetimes of 110+/−44 s, compared to 51+/−16 s for pits lacking virus (Figure 1F). Thus, efficient internalization of VSV occurs through coated pits containing AP-2, and compared with pits lacking virus, those containing virus have increased lifetimes and often contain more clathrin and AP-2.

**Figure 2:**
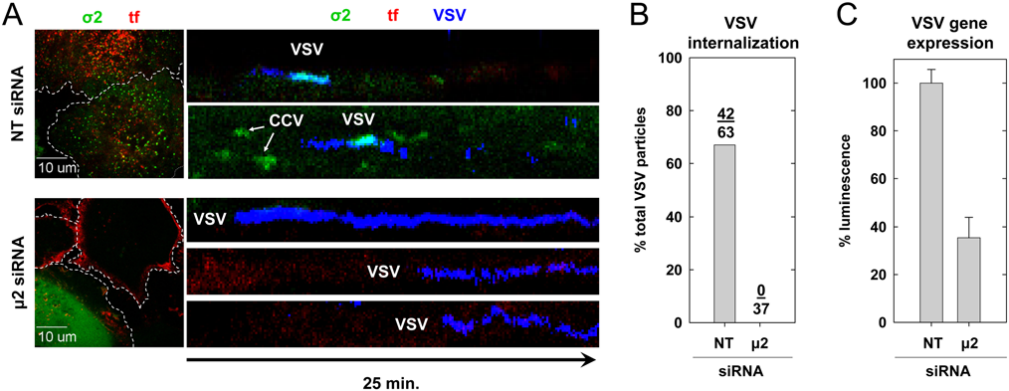
AP-2 is a functional adapter for VSV internalization. (A) An image of BSC-1 cells (left) stably expressing σ2-eGFP (green) treated with non-targeting (NT) or μ2-adaptin siRNAs and exposed to Alexa 568 labeled tf (red). Images were acquired from the bottom and middle of the NT and μ2 siRNA-treated cells, respectively. Dashed white lines demark the cell boundaries. A series of kymograph views showing VSV (blue) internalization in siRNA treated cells (right). Note the lack of virus internalization in cells lacking σ2-eGFP. (B) A graph of the % of bound VSV particles that were internalized by 7 cells treated with the NT siRNA and 5 cells treated with the μ2 siRNA and defective for transferrin uptake. (C) A graph depicting the effect of μ2 depletion on VSV gene expression. Cells were doubly transfected with the indicated siRNAs and inoculated with rVSV-LUC at an MOI of 0.5. Virus particles were removed after 1 h, and luminescence was quantified at 4 h p.i. Values represent the mean+/−the standard deviation of 2 independent experiments.

Using siRNA to target the μ2 subunit of AP-2 in cells constitutively expressing σ2-eGFP, we showed that AP-2 depletion completely inhibited clathrin-dependent VSV internalization (Figure 2). Loss of μ2 can be followed by disappearance of the σ2-eGFP signal from coated pits, as assembly of one AP-2 heterotetramer depends on all four of its subunits. As expected, cells lacking detectable σ2-eGFP (>95% of the population) failed to take up an AP-2 dependent cargo, transferrin, which remained trapped at the cell surface (Figure 2A, red signal). Moreover, inspection of 37 virus particles attached to 5 different cells depleted for AP-2 showed that VSV remained attached at the cell surface (Figure 2A, blue signal). By contrast, cells treated with a control siRNA displayed normal levels of σ2-eGFP, robust transferrin uptake, and maintained the capacity to internalize VSV (42/63 particles; 8 cells) (Figure 2A, B). In other cells, traces of AP-2 expression remained following siRNA treatment. Under such conditions, we found that low levels of AP-2 accumulated beneath particles, and that virus was internalized by clathrin (not shown). Thus, low levels of AP-2 are sufficient for clathrin-dependent virus uptake. Using a recombinant VSV that expresses firefly luciferase as a marker of infection (rVSV-LUC), we further show that μ2 depletion resulted in diminished infection of cells as indicted by a 65% reduction in luciferase activity (Figure 2C). These data demonstrate that AP-2 is essential for the clathrin-dependent uptake of VSV.

### Dynamin is required for VSV entry

**Figure 3:**
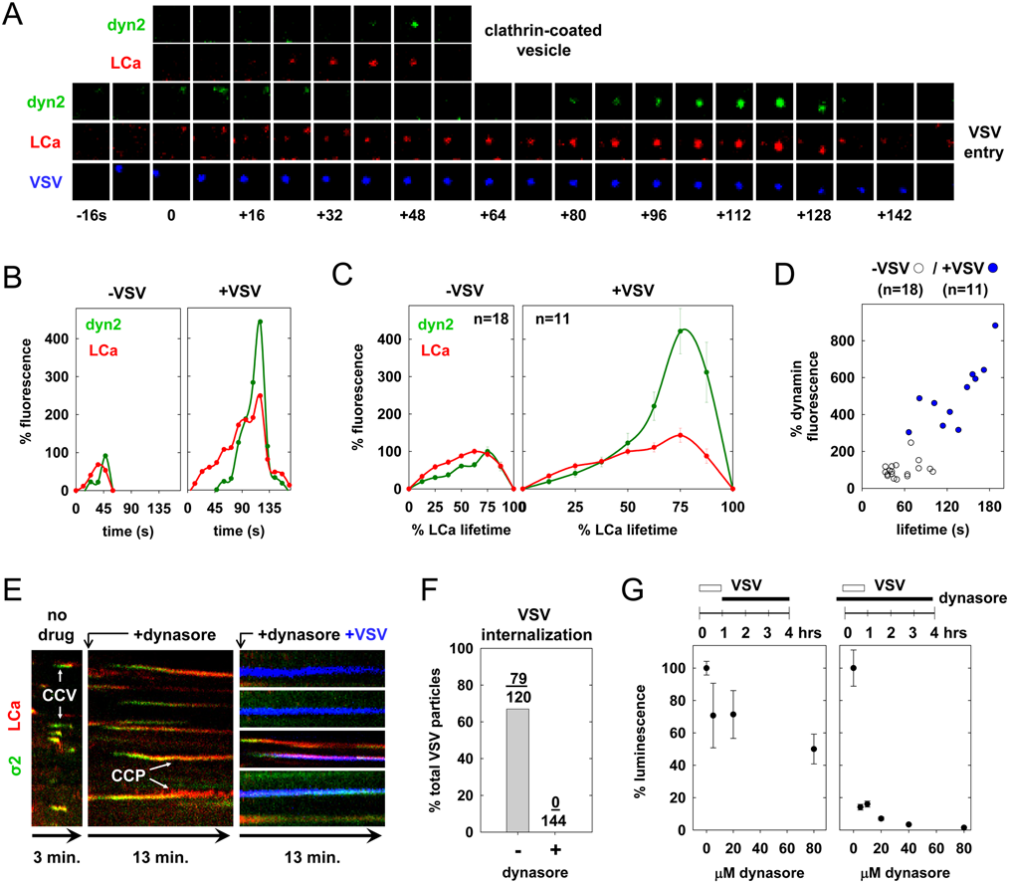
Enhanced dynamin recruitment during viral internalization. (A) A tile view of images from a BSC-1 cell co-expressing dyn2-eGFP (green) and tom-LCa (red) cropped from a time-lapse movie (Video S3), showing appearance of VSV at the cell surface (t = −8 s) relative to detection of clathrin (t = 0 s). (B) A graph of the kinetics of dyn2 and clathrin recruitment to the CCPs shown in panel A. The fluorescence was expressed as a % of the average maximum clathrin measured in all pits lacking virus, and a weighted average was plotted as described in Materials and Methods. (C) A graph of the average kinetics of LCa and dyn2 recruitment to CCP containing (right) or lacking (left) virus. Average fluorescence intensity and time are expressed as a % relative to CCV lacking virus observed in the same cells. Fluorescence intensity was calculated at 8 equally-spaced intervals and is plotted+/−standard error. Viral events were from 5 cells, and events lacking virus were analyzed in 3 of the same cells. (D) A graph of the maximum dynamin recruitment relative to pit lifetime for CCVs containing (blue) or lacking (open circles) virus. Dynamin fluorescence is expressed as a % of the average peak observed for pits lacking virus. The average lifetime of pits containing virus was 148+/−71 s (5 cells), and was statistically distinct (Student's t-test: p = 7e-4) to that for pits lacking virus (55+/−21 s from 3 of same cells). The peak dynamin fluorescence intensities in pits containing and lacking virus were 548+/−221 and 100+/−45, respectively. The difference between these values is statistically significant (Student's t-test: p = 2e-5). (E) A kymograph view of BSC-1 cells expressing σ2-eGFP and tom-LCa. Note the lack of CCP internalization (arrows) and virus (blue) uptake in cells treated with 80 µM dynasore. Video S4 depicts inhibition of VSV internalization following dynasore treatment. (F) A graph of the % of bound VSV particles that were internalized in the presence (n = 4 cells) and absence (n = 5 cells) of dynasore. (G) Graphs of the effect of dynasore on VSV entry and gene expression. Cells were infected with rVSV-LUC (MOI = 0.5), and exposed to the indicated concentration of dynasore 1–4 h p.i. (left) or prior to infection −0.5–4 h p.i. (right). Luminescence values were measured at 5 h p.i. and are the average+/−standard deviation of triplicate samples.

Using cells expressing low levels of dynamin2-eGFP (dyn2-eGFP) and tom-LCa, we compared dynamin recruitment to 11 pits containing virus with 18 that lacked virus. As previously demonstrated for pits lacking virus, dynamin recruitment increased rapidly during late stages of pit growth (Figure 3A–C). Virus-containing pits also recruit dynamin, with a spike during the later stages of growth (Figure 3A–C, Video S3). Quantitation of the peak dyn2-eGFP fluorescence shows that virus-containing pits recruit an average of five times more dynamin than pits lacking virus (Figure 3C, D).

Using dynasore [10], a cell permeable small molecule inhibitor of dynamin, we showed that dynamin function is required for clathrin-dependent internalization of VSV. As previously reported [10], addition of dynasore caused arrest of coated pit formation and trapped clathrin structures at the plasma membrane (Figure 3E). Clathrin-dependent uptake of VSV was likewise completely inhibited (Figure 3E, F). Virus particles that entered coated pits within the first minute following dynasore addition failed to internalize and remained trapped for the 13 min. duration of the time-lapse video in structures that contained clathrin and AP-2 (Figure 3E and Video S4). Of 144 virus particles that attached to cells in the presence of dynasore, none was internalized by clathrin (Figure 3F), in contrast to the highly efficient clathrin-dependent internalization of VSV in the absence of dynasore (Figure 3F). Using the rVSV-LUC assay, we also showed that dynasore functionally inhibits viral entry (Figure 3G). VSV gene expression was relatively insensitive to the addition of dynasore at 1 hour post-inoculation but not to its addition just prior to virus inoculation (Figure 3G). These data show that functional dynamin is critical for clathrin-dependent uptake of VSV and concomitant productive infection of cells.

### Auxilin recruitment to vesicles containing VSV

**Figure 4:**
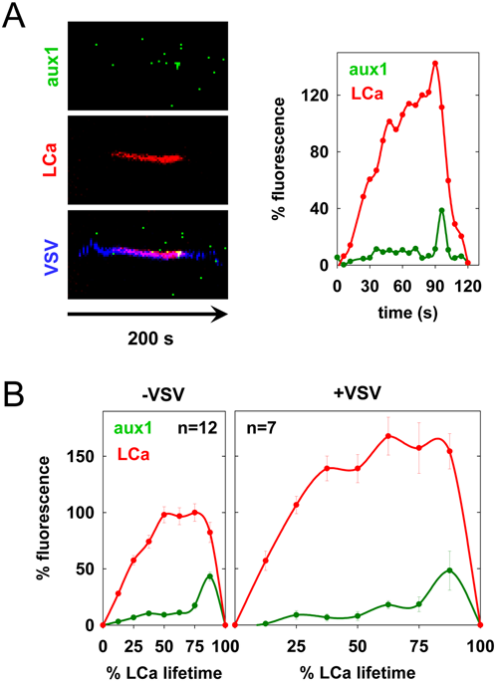
Auxilin recruitment during uncoating of vesicles containing VSV. (A) Kymograph views of VSV internalization by a BSC-1 cell expressing tom-LCa and eGFP-aux1 (left), along with a graph of the kinetics of auxilin (aux1) and clathrin (LCa) recruitment (right). The internalization event shown is depicted in Video S5. (B) Graphs of the average kinetics of clathrin and auxilin recruitment to CCP containing (right) or lacking (left) virus. Average fluorescence intensity and time are expressed as a % relative to CCV lacking virus observed in the same cells. Fluorescence intensity was calculated at 8 equally-spaced intervals and is plotted+/−standard error. Viral events were from 2 independent cells, and events lacking virus were analyzed in 1 of these cells. The difference between the maximum aux1 fluorescence in pits containing and lacking VSV is not statistically significant (Student's t-test: p = 0.1).

The cellular ATPase, Hsc70, and its cofactor, auxilin, catalyze clathrin disassembly from endocytic vesicles. Auxilin1 is recruited to coated pits upon completion of clathrin growth at the time of vesicle budding, triggering rapid uncoating of the newly formed vesicle [4],[5]. Using cells co-expressing eGFP-aux1 and tom-LCa, we examined whether coated pits that contain virus also recruit auxilin. We acquired images at 2-second intervals to ensure that we captured the full kinetics of auxilin arrival. We imaged the internalization of 7 viral particles and compared the lifetimes and abundance of tom-LCa and eGFP-aux1 in these vesicles with 12 that lacked virus. The extent of auxilin1 recruitment to coated pits was unaltered by the presence of VSV (Figure 4A, Video S5). By measuring the duration of the spike in eGFP-aux1 fluorescence, we found a slight but statistically insignificant increase in the duration of the auxilin burst from 5+/−1 s to 7+/−2 s during VSV entry. These data show that virus-containing vesicles uncoat similarly to those that lack virus (Figure 4B).

### Local actin polymerization during VSV internalization

**Figure 5:**
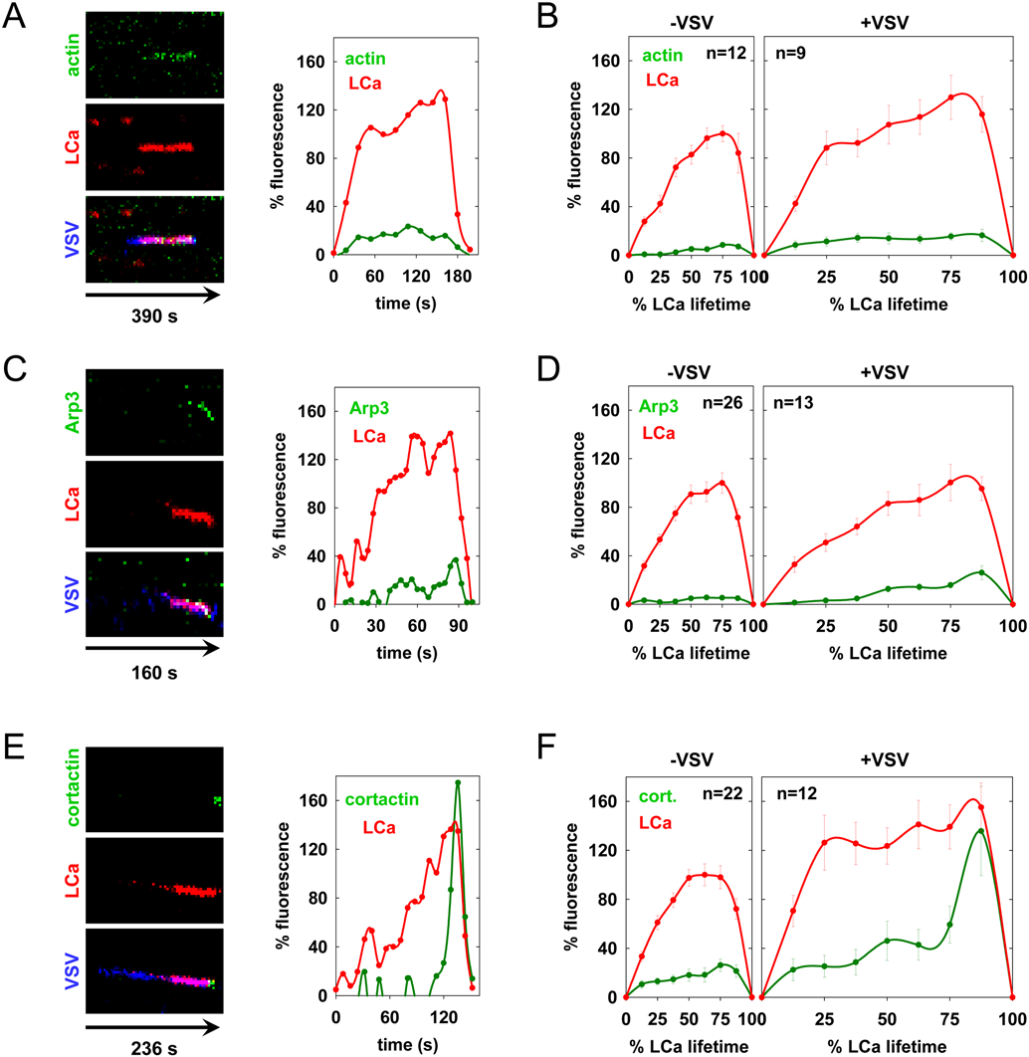
Actin cytoskeletal dynamics during clathrin-dependent uptake of VSV. (A, C, and E). VSV internalization events in BSC-1 cells co-expressing tom-LCa and eGFP-actin (A), arp3-eGFP (C), or cortactin-eGFP (E) are shown as kymographs and in Videos S6, S7, S8 respectively. The fluorescence over time (left to right) of the actin cytoskeletal component (top), tom-LCa (middle), and a merge of these traces with the virus are shown (bottom) and are represented graphically at the right using the same approach as in Figure 1D. (B, D, and F) Graphs of the average kinetics of clathrin and actin (B), arp3 (D) and cortactin (F) recruitment to vesicles lacking (left) or containing (right) virus are shown. Average fluorescence intensity and time are expressed as a % relative to CCV lacking virus observed in the same cells. Fluorescence intensity was calculated at 8 equally-spaced intervals and is plotted+/−standard error. Virus internalization data was collected from 5, 3, and 2 cells for panels B, D and F respectively, and compared with events lacking virus from 2, 2, and 3 of the same cells, respectively. The differences between the maximum actin, Arp3, and cortactin fluorescence values in pits containing and lacking VSV are statistically significant. Student's t-test: actin p = 2e-5; Arp3 p = 4e-4; cortactin p = 0.003.

The effects of VSV on clathrin-coated pit formation suggest parallels with internalization of large and more stable clathrin-coated structures (Saffarian and Kirchhausen, *submitted*) and the invasive bacteria *Listeria monocytogenes* [11]. Actin polymerization has a role in the internalization of such structures, although it is not required for internalization of conventional clathrin-coated pits [12] and Saffarian and Kirchhausen, *submitted*. We therefore tested whether actin, an actin nucleation factor, Arp3, or cortactin, an activator of the Arp2/3 complex, is recruited to coated pits. We used for this purpose, cells expressing tom-LCa and eGFP fused to each of these proteins. During growth of virus-containing pits, the levels of actin, Arp3, and cortactin increased steadily (Figure 5B, D and F). Moreover, for both Arp3 and cortactin, there was a peak of fluorescence just prior to the onset of vesicle uncoating (Figure 5A–F, Videos S6, S7, S8). In contrast, pits that lacked VSV had low and variable levels of actin and Arp3 fluorescence throughout the growth phase that were barely above the background (Figure 5B and D). Cortactin recruitment was approximately 4-fold lower than observed in virus-containing pits (Figure 5F). We conclude that actin assembly is associated with VSV entry through a clathrin pathway.

### Inhibition of actin polymerization reduces the efficiency of VSV internalization

**Figure 6:**
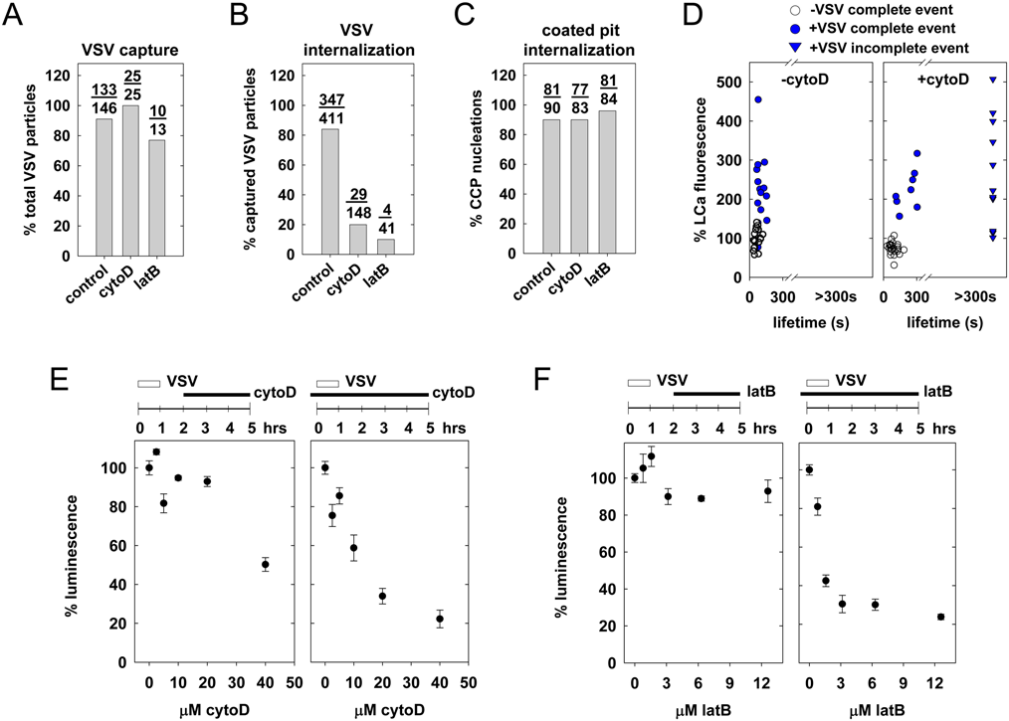
Chemical inhibition of actin polymerization reduces the efficiency of VSV internalization. (A–D) Where indicated, BSC-1 cells were treated with 20 µM cytochalasin D (cytoD), or 6.3 µM latrunculin B (latB) for 10 min. prior to inoculation with VSV and acquisition of time-lapse images. Video S9 depicts the effect of cytoD treatment on VSV internalization. The % of attached particles captured by a coated pit (A), and subsequently internalized (B), along with the % of CCP lacking virus that complete within 80 s (C), were plotted to generate the graphs shown. Data in (A) and (B) are from 28 independent control cells, 5 cells treated with cytoD, and 2 cells treated with latB. Data in (C) are from 2 independent cells in each condition. (D) The graphs show clathrin fluorescence vs. CCV lifetime for complete (circles) or incomplete (triangles) internalization events, for pits containing (blue) or lacking (open circles) VSV. Clathrin fluorescence is expressed relative to its intensity in untreated pits lacking virus, which were set to 100. Internalization was analyzed from 4 cells in the absence of cytoD (16 complete VSV internalizations, 23 complete non viral events) or 3 cells (8 complete and 11 incomplete VSV events, 24 complete non viral events) in the presence of cytoD. The peak clathrin fluorescence in viral events was not statistically significantly different in the presence and absence of cytoD (Student's t-test p = 0.2 for complete and incomplete events). (E) Effect of cytoD on VSV entry and gene expression. Cells were infected with rVSV-LUC (MOI = 0.5) and exposed to the indicated concentration of cytochalasin D 2–5 h p.i. (left) or prior to infection −0.5–5 h p.i. (right). Luminescence values were measured at 5 h p.i. as described in Materials and Methods. The values shown are the mean of triplicates+/−standard deviation. (F) Effect of latB on VSV entry and gene expression. Cells were treated as in (E) except latB was substituted for cytoD.

To assess the importance of actin function during VSV internalization, we used cytochalasin D (cytoD) or latrunculin B (latB) to inhibit actin assembly. CytoD binds to the growing ends of actin filaments [13], and latB sequesters G-actin monomers [14]. As shown previously [12], cytoD or latB diminishes the number of clathrin-coated pit nucleation events, but has no effect on either the kinetics of coat assembly or the subsequent internalization of these pits (Figure 6C). Pretreatment of cells with cytoD or latB did not affect the efficiency (Figure 6A) or time (mean = 160 s; Student's *t*-test p = 0.24) to association of virus with a clathrin-coated pit. Instead, both compounds inhibited entry by blocking by at least 75% the transition of virus-containing pits to completed vesicles (Figure 6B).

We further established that treatment with cytoD, which has little detectable effects on the kinetics of virus-free coated pit formation, freezes assembly of virus-containing clathrin coats (Figure 6D). In the presence of cytoD, the increase in clathrin fluorescence at puncta labeled by VSV reached a level similar to the average level for untreated cells, but the two labels then remained associated, often for the duration of the experiment (Figure 6D, Video S9). Those virus particles that did internalize (as detected by loss of clathrin signal and rapid motion of virus label away from the site of coated-pit formation) remained associated with clathrin for 263+/−34 s, albeit 3 times longer than the mean life time of virus-free coated pits (Figure 6D). Using rVSV-LUC, we also showed that treatment with cytoD and latB during infection had a dose-dependent effect on luciferase gene expression, while introduction of these compounds at 1 hour post-infection had little effect for over three hours following addition of the drug (Figure 6E, F). We conclude that actin assembly is critical for internalization of VSV but not for gene expression once virus has entered. Moreover, at least one actin-dependent step is essential for completion, pinching and dissociation of the clathrin coat, but not for its nucleation and initial assembly.

### Clathrin assembly is locally induced by VSV particles

**Figure 7:**
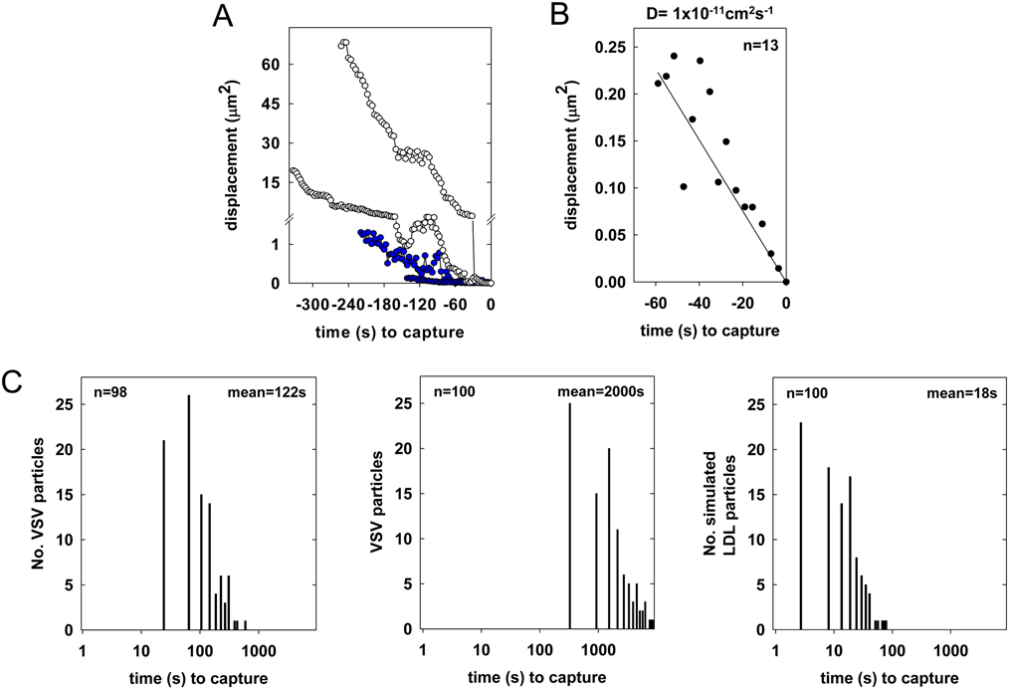
Kinetics of VSV diffusion and capture by clathrin-coated pits. (A) A graph of the diffusion (mean squared displacement) of 4 single particles relative to the time of clathrin detection (t = 0) is shown. Note the break in the Y axis to emphasize the difference between rapidly (open circles) and slowly (blue) diffusing particles. (B) A graph of the average rate of diffusion (mean square displacement) for 13 particles (n = 4 cells) tracked for 60 s prior to clathrin detection (t = 0) is shown. (C) A histogram of the time between VSV attachment and the appearance of clathrin or adaptor is shown for 98 particles (left; n = 28 cells), and compared with the simulated kinetics of random capture of VSV (middle) or LDL (right) by CCPs. The mean times to cargo capture are shown in the upper right corner of each panel.

Is capture of VSV by a coated pit a random diffusional collision as it is for LDL [1], or does the virus induce coat formation after binding? We studied the trajectories of 37 virus particles at the cell surface and found that most (n = 25) diffused randomly (Figure 7A, B), with diffusion coefficients (D) between 5×10^−11^ cm^2^ s^−1^ and 5×10^−12^ cm^2^ s^−1^. A subset of particles moved more rapidly (average D = 5×10^−10^ cm^2^ s^−1^) but eventually also slowed to comparable diffusion rates (Figure 7A). Regardless of the initial diffusion rate, all viral particles became essentially immobile at least 20 s (mean = 42 s) prior to the earliest detection of clathrin (Figure 7A, B). For the subset of particles that attached during imaging and then entered productively (n = 98), the average time between virus binding and clathrin detection was 122 s (Figure 7C). Analysis of all virus capture events showed that clathrin colocalized with particles from the moment of its detection, indicating that pits that capture VSV form close to or directly beneath virions.

To test whether our experimental observations are consistent or not with a random capture of VSV by clathrin, we performed a Monte-Carlo simulation. We used MATLAB to approximate the random motion of VSV particles on a 100×100 grid with the experimentally determined diffusion rate of 1×10^−11^ cm^2^ s^−1^. We estimated a viral footprint of 120×120 nm^2^, which is a generous estimate of the surface area directly contacted by a virus of 180×70 nm. We introduced clathrin-coated pits with lifetimes of 20 s (the time beyond which a pit would be too constricted to accommodate a VSV particle) at random locations according to the experimentally determined nucleation rate of 0.6 pits/10^8^ nm^2^ s^−1^, which was derived from 6 cells. To estimate the diffusion coefficient, we calculated the typical rate of particle displacement by plotting the square of the mean displacement versus time (Figure 7B). The slope of the line of best-fit provides a rate of 0.0041 µ^2^ s^−1^, which we used to calculate a representative diffusion coefficient (D) of 1×10^−11^ cm^2^ s^−1^ using the formula: (mean displacement)^2^ = 4Dt. This most accurately reflects the rate of virus diffusion at the time of capture (Figure 7B). Using these parameters, the average time to virus capture was 2000 s, which is markedly different to the measured time of 122 s (Figure 7C). Our simulation predicts a mean capture time of 18 seconds for an LDL particle, which is in good agreement with the experimental value of 20 seconds [1]. This validates our simulation protocol, and together with the virus data, provides evidence that pits engaged in virus uptake preferentially nucleate in close proximity to VSV particles.

## Discussion

We examined how VSV enters host cells via the clathrin-dependent endocytic machinery using a combination of high-resolution live cell imaging, electron microscopy and quantitative assays for viral infectivity. By comparing clathrin-dependent uptake of single virus particles with canonical coated vesicle formation, we uncovered striking differences between these two processes. Unlike typical coated vesicles, virus-containing vesicles lacked a full clathrin coat and required local actin polymerization after coat assembly for efficient internalization. We also obtained evidence that VSV promotes its own uptake by inducing clathrin coat formation in close proximity to the virus particle. This study uncovers one mechanism by which mammalian cells internalize large clathrin-dependent cargo through the coordinated action of endocytic and cytoskeletal components. This mode of clathrin-dependent endocytosis shares some features of the clathrin endocytic machinery of *Saccharomyces cerevisae*, as well as the uptake of large bacteria such as *Listeria monocytogenes* into mammalian cells.

### VSV internalization through partially clathrin-coated structures

**Figure 8:**
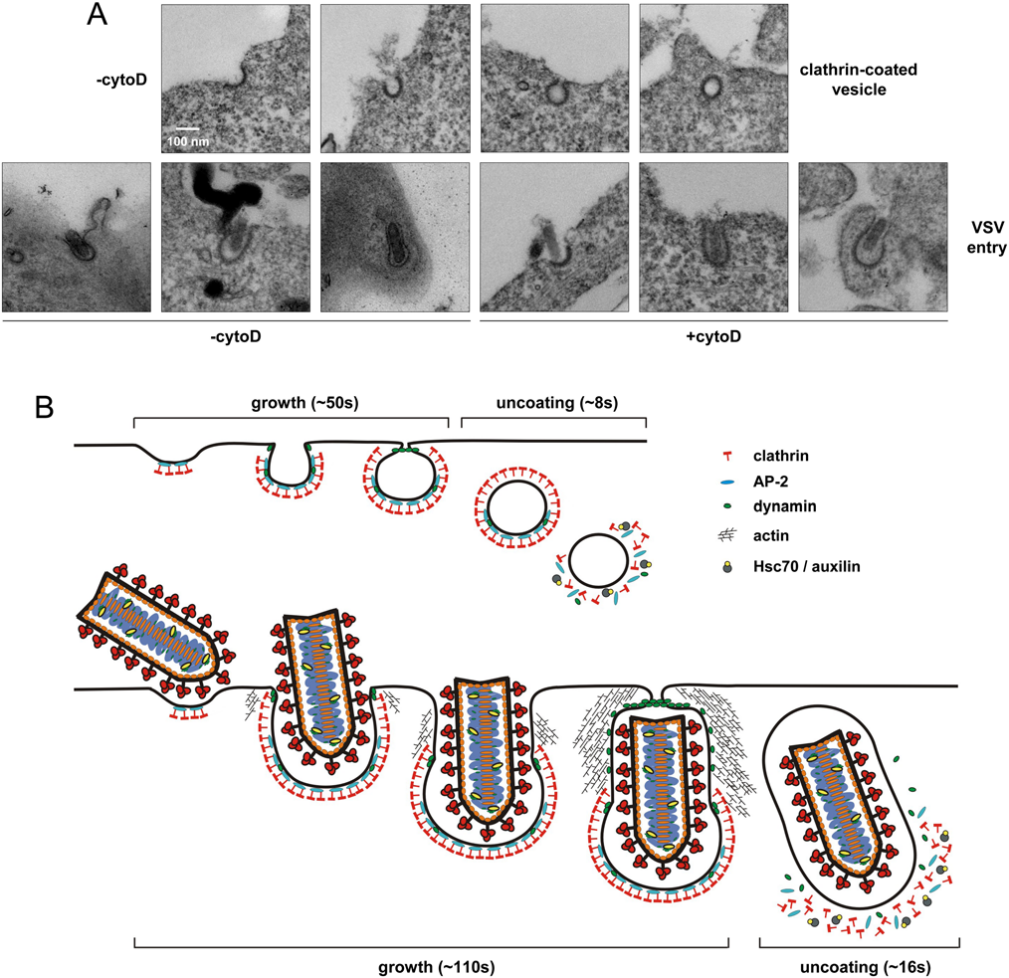
Visual comparison of CCV formation and VSV entry. (A) Electron micrographs depicting successive stages of CCV formation (top) and VSV entry (bottom). Clathrin appears as an electron dense coat on the cytosolic side of the plasma membrane, and early stages of clathrin assembly are indistinguishable. Conventional pits then adopt a constricted U shape, while virus-containing structures form elongated clathrin-coated tubes. Pits lacking virus become fully coated and pinch off from the membrane as spherical vesicles, but viral pits mature into much larger, partially uncoated structures. In the indicated samples, cells were treated with 20 µM cytoD for 10 min., and incubated with rVSV at an M.O.I. of 5000 for 15 min. Cells were fixed and stained as described in Materials and Methods. (B) Model of VSV internalization by the clathrin and actin machinery. Conventional clathrin-coated vesicles (top) constitutively nucleate on the cell surface and grow by addition of clathrin and adaptor proteins until a fully-coated, constricted pit is formed. Pits are severed from the plasma membrane in a dynamin-dependent, but actin-independent manner, and the clathrin coat is rapidly disassembled by Hsc70 and auxilin. During clathrin-dependent internalization of VSV, pits preferentially form in close proximity to virus particles. The growing clathrin lattice imparts a curvature to the tip of the pit, but coat assembly ceases when the constricted edge of the pit meets the enclosed particle. The actin cytoskeletal machinery is then recruited by dynamin to drive further invagination of the particle. Dynamin, possibly in conjunction with actin, mediates fission of the virus-containing pit, and clathrin is uncoated.

We previously established the direct relationship between coat size and clathrin or AP-2 fluorescent intensity associated with endocytic coated pits and vesicles [1]. We compared the amount of clathrin associated with vesicles containing and lacking VSV. On average, virus-containing vesicles contained 1.5-fold more clathrin, with a maximum of 2.5-fold more, and in some instances, less clathrin than vesicles lacking virus (Figure 1F). Although pits internalizing VSV tend to have more clathrin, the increase is insufficient to coat fully a vesicle containing a viral particle. The size of VSV particles is 180×70 nm, which could fit within a spherical vesicle with an internal radius of 90 nm, or within a prolate spheroid with a polar radius of 90 nm and an equatorial radius of 35 nm. The surface area of such vesicles is respectively 9 and 3 times greater than that of a conventional clathrin-coated vesicle with an internal radius of 30 nm. Thus to be completely coated with clathrin, virus-containing vesicles would require at least 3 times the amount of clathrin observed on a conventional vesicle. Our data are not consistent with this level of clathrin on vesicles internalizing VSV, which leads us to conclude that clathrin does not fully coat such vesicles. Further support for this conclusion is provided by electron micrographs that show VSV present within clathrin-coated structures that contain an elongated tubular neck that lacks the characteristic clathrin density (Figure 8A). These images are also consistent with earlier electron micrographs that show VSV entering cells where clathrin is apparent on only a portion of the vesicle [15],[16],[17]. However, it was uncertain from this earlier work as to whether the static EM images represent an intermediate stage of the clathrin assembly process during virus internalization. By examining this process in real time in live cells, our data provides compelling evidence that virus enters cells in vesicles that lack a full clathrin coat.

### Clathrin adaptor usage during virus internalization

The heterotetrameric AP-2 adaptor complex recruits clathrin triskelions to the plasma membrane through direct interactions with phospholipids, receptor molecules, and clathrin. Almost all clathrin-coated pits at the cell surface contain AP-2, and depletion of its μ2 subunit reduces pit formation >10-fold [18]. Functionally, AP-2 depletion inhibits transferrin uptake [18]; however, its effect on other clathrin-dependent cargo is less clear. For example, depletion of the σ2 subunit of AP-2 inhibits EGF uptake at 37°C [19] but not after preincubation of cells with the ligand at 4°C [18],[19],[20]. For influenza A virus, depletion of epsin 1 [21], but not the α-subunit of AP-2 [20], impaired the clathrin-dependent internalization of virus particles. Following submission of this manuscript, a related study of the internalization of VSV reported that depletion of the α- and μ-subunits of AP-2 in HeLa cells did not inhibit infection [22]. However, the authors were unable to directly image the viral entry step and thus could not determine whether the virus entered the AP-2 depleted HeLa cells in a clathrin-dependent or independent manner. In the present study, we found that depletion of μ2-adaptin in BSC-1 cells prevents clathrin-dependent uptake of VSV and diminishes viral VSV gene expression (Figure 2). In BSC-1 cells simultaneously expressing σ2-eGFP and tom-LCa, AP-2 ablation reduced the rate of coated vesicle formation by ∼10-fold, and pits that formed contained low levels of AP-2. Importantly, VSV was only internalized by clathrin containing structures that also contained σ2-eGFP fluorescence. Thus, our data supports an essential requirement for AP-2 in the clathrin-dependent uptake of VSV, at least in BSC-1 cells. Why the clathrin-dependent endocytosis of influenza A virus is insensitive to AP-2 depletion and appears to depend on Epsin 1 is not clear. Perhaps this distinction reflects differences in cellular receptor usage or the capacity to trigger recruitment of specific cell factors to sites of virus binding.

### The actin machinery plays a key role in virus internalization

Although much evidence supports a role for the actin machinery in clathrin-dependent endocytosis in the yeast *Saccharomyces cerevisae*, its role in mammalian cells is uncertain. In *S. cerevisae*, clathrin-coated patches on the cell surface mature into ∼200 nm tubular invaginations with coat proteins at their tip [23]. The actin cytoskeletal machinery is recruited after coat formation and provides an essential force for membrane deformation and internalization [24]. In mammalian cells, clathrin structures differ in their actin dependency for internalization. Chemical inhibitors of actin polymerization reduce the rate of clathrin-coated pit formation by 2-fold but do not block the uptake of conventional coated pits [12]. Live cell imaging studies have provided evidence that a subset of clathrin structures assembled at the plasma membrane recruit the actin machinery and require actin function for their internalization [12],[25],[26],[27],[28]. What distinguishes this subset of structures is uncertain, but they may correspond to large arrays of clathrin, as their lifetimes are much longer (>60 s) than that of conventional coated pits.

In the present study, we found that clathrin structures internalizing VSV require actin polymerization for efficient uptake into cells (Figure 6). Analogous to the situation in yeast [24],[29], actin polymerization is required downstream of clathrin recruitment (Figure 6D). Precisely what step of internalization is actin-dependent is uncertain, but perhaps actin filament formation facilitates the invagination and possibly fission of coated pits containing a VSV particle. Our findings of a requirement for the actin machinery in VSV internalization contrast with earlier work, which showed that VSV infection of polarized MDCK cells from the apical but not from the basolateral surface was inhibited on treatment of cells with cyto D [30]. In those experiments, however, the basolateral uptake of luciferase yellow, a general tracer for all forms of internalization, was not affected. Thus, basolateral entry of VSV through a clathrin/actin-independent route can explain the successful infection of cytoD-treated MDCK cells, and future direct imaging studies could establish the entry route.

What regulates actin polymerization during VSV entry is uncertain. However, we did not observe global changes in the actin cytoskeleton during viral entry, indicating that this must occur locally. Perhaps a physical association between components of the actin and clathrin machineries regulates local actin polymerization during virus uptake. One possible candidate that could provide this association is dynamin, which contains binding sites for several proteins that directly or indirectly activate the Arp2/3 complex, including cortactin [31],[32], syndapin [33],[34], and intersectin [35],[36]. Consistent with this possibility, the recruitment of dynamin is coincident with the recruitment of cortactin during the late stages of virus internalization (Figures 3, 5). Further investigation will be required to fully understand the requirement for actin function during VSV entry.

### VSV induces its own uptake by clathrin-coated pits

Transferrin and LDL-receptor complexes diffuse rapidly on the plasma membrane prior to their capture by pits, which constitutively sample areas of the cell surface [1]. Here we measured the diffusion of VSV particles and show that they diffuse 60 times slower than LDL-receptor complexes (Figure 7B). However, the 122 s average time between binding of VSV to the cell and its capture by a pit is only 6-fold longer than the measured time to capture LDL [1]. Using a simulation that accurately predicts the average capture time of LDL as 20 s, we estimate that random capture of VSV requires 2000 s (Figure 7C). How might we account for this substantial difference between the predicted and observed time to VSV capture? We tested how adjusting the parameters used in our simulation, which accurately predicts the time for LDL capture, would alter the predicted time to VSV capture. Biologically relevant adjustments to the rate of virus diffusion or the density of pit formation on the plasma membrane are unable to generate a predicted time to virus capture that is close to the measured value of 122 s (Figure S2A). Our data further show that the difference between the observed and predicted time to capture does not reflect the time taken to visualize a coated pit. If we assume that most conventional clathrin-coated vesicles contain 60 clathrin triskelions (consistent with a soccer ball shape), the peak fluorescence associated with such vesicles indicates that 7 triskelia are sufficient for detection. The average lifetime of a conventional vesicle is 50 seconds, indicating that pits may initiate up to 6 seconds before we can visualize them. Finally, adjustments to the assumed footprint of the virus on the plasma membrane ranging from the area contacted by a single G trimer to the entire virus particle cannot account for the rapid virus uptake (Figure S2B). Thus, adjusting any of the parameters of our simulation fails to approximate the observed time to virus uptake. We therefore propose that VSV induces its own uptake by the clathrin machinery. Precisely how VSV particles can influence pit nucleation is unknown. Earlier studies measured the time between attachment and capture for reovirus [1] and influenza A virus [9] particles by coated pits. Influenza particles were captured after an average of 190 s, while reovirus remained on the cell membrane for 280–1500 s before associating with clathrin. Although the basis for the different uptake times of these viruses is unknown, it may reflect properties inherent to the virus particles themselves or the engagement of specific receptors during entry.

### Model for the clathrin and actin-dependent internalization of VSV

Our data support the following model of VSV entry by clathrin-dependent endocytosis (Figure 8B). Virus interaction with the cell surface promotes its own uptake by the clathrin machinery. The clathrin machinery appears to nucleate in close proximity or directly beneath the virus particle. The coated pit grows at a steady rate that is similar to a conventional one, except that the growth phase is prolonged, perhaps reflecting the larger size of the viral cargo. We suggest that the clathrin coat is unable to encircle completely the virus particle, at least in part because the virus serves as physical barrier to closing the pit. This inhibits the incorporation of additional clathrin molecules into the coat leading to the formation of a partially coated structure with an elongated neck (Figure 8B). We propose that actin polymerization facilitates the invagination of this partially coated pit by generating a force that helps lift the membrane away from the vesicle or pushes the vesicle into the cell (Figure 8B). The process of scission may be accomplished by a mechanochemical activity of dynamin. More dynamin may be required for this process, and consistent with this idea, virus-containing pits recruit increased quantities of dynamin. The separated pit is then rapidly uncoated in a manner that depends upon auxilin and appears similar to that of conventional vesicles.

### Parallels with the clathrin-dependent uptake of bacteria

Our findings with VSV also share parallels with the clathrin-dependent uptake of *L. moncytogenes*. Specifically, clathrin, dynamin, auxilin, and actin are recruited during entry of both pathogens, and actin polymerization is critical for their endocytosis but not the clathrin assembly process [11],[37]. In addition, localization of the actin machinery to sites of *Listeria* invasion appears to require the prior recruitment of clathrin and dynamin [11]. The nature of this linkage between the clathrin and actin systems is unknown, but perhaps VSV and *Listeria* also utilize similar mechanisms to coordinate actin polymerization during their uptake [11],[37]. Despite these commonalities, the adaptor protein involved in *L. monocytogenes* entry is unclear, as neither AP-1, -2, nor -3 accumulate [11]. This may reflect differences in receptor usage or differences inherent to the clathrin structure that forms.

Our observations demonstrate a requirement for the actin machinery in the clathrin-dependent uptake of VSV. It will be important to determine if linkage of coated pits to the actin cytoskeleton is a general response of cells to the presence of large clathrin-coated structures or whether the interaction is triggered in a receptor-specific manner. Bacterial pathogens such as *L. monocytogenes* and *Staphylococcus aureus* induce recruitment of the clathrin machinery through the interaction of bacterial surface proteins with their cognate cellular receptors [11]. Whether viruses share this capacity is unknown, but several viruses, including SV40 [38], poliovirus [39],[40], group B coxsackievirus [41], and vaccinia virus [42] are known to promote their own uptake in a clathrin-independent manner.

## Materials and Methods

### Cells and virus

BSC-1 cells (ATTC CCL-26) were maintained in DMEM (Invitrogen Corporation; Carlsbad, CA) supplemented with 10% fetal bovine serum (Tissue Culture Biologicals; Tulare, CA). Cells stably expressing σ2-eGFP [1] were maintained as above in the absence of selective agent. Recombinant VSV (rVSV) [43] and rVSV eGFP-P [44] were amplified in BHK-21 cells (ATTC) and purified on linear 15–45% sucrose gradients prepared in NTE (10 mM Tris pH 7.4, 100 mM NaCl, 1 mM EDTA). Concentrated virus stocks were stored in PBS containing 10 mM HEPES (pH 7.4) at −80°C. A recombinant VSV, rVSV-LUC, expressing firefly luciferase was generated by insertion of the luciferase coding region flanked by the conserved VSV gene-start and -end sequences between the leader and *N* gene. Virus was recovered from cDNA as described [43].

### Dye conjugation to VSV particles

Alexa Fluor 647 (Molecular Probes, Invitrogen Corporation) was solubilized in DMSO at 10 mg/mL and incubated at a final concentration of 62.5 µg ml^−1^ with purified VSV (1 mg ml^−1^) in 0.1 M NaHCO_3_ (pH 8.3) for 90 min. at room temperature. Virus was separated from free dye using a NAP-5 gel filtration column (GE Healthcare; UK), eluted in PBS containing 10 mM HEPES (pH 7.4) and stored at −80°C. To measure the effect of labeling on virus infectivity, equivalent amounts of total viral protein from labeled and unlabeled preparations were titrated by plaque assay on Vero cells.

### Nucleic acid transfection

Approximately 60,000 BSC-1 cells were seeded on glass coverslips (25 mm diameter, no. 1.5, Electron Microscopy Sciences; Hatfield, PA) 16–20 h prior to transfection with the indicated plasmid. Parental BSC-1 cells or BSC-1 cells stably expressing rat σ2-adaptin [1] were transfected with 0.5 µg of plasmid encoding either rat brain clathrin light chain A1 fused to the tandem repeat tomato open reading frame (tom-LCa, [4]), or rat dynamin2-eGFP (kind gift of Dr. Sandra Schmid; The Scripps Research Institute, La Jolla, CA), or bovine eGFP-auxilin1 [4], or mouse cortactin-eGFP (kind gift of Dr. David Drubin; The Department of Molecular and Cell Biology, University of California, Berkeley, CA) [45],[46], or human arp3-eGFP (kind gift of Dr. Timothy Mitchison; Department of Systems Biology, Harvard Medical School, Boston, MA) [47], or human eGFP-β-actin (pAcGFP1-actin; Clonetech Laboratories, Inc.; Mountain View, CA) [25],[48] using FUGENE 6 according to the manufacturer's instructions (Roche Diagnostics; Indianapolis, IN). Cells were imaged 20–24 h post-transfection as described below.

Where indicated, prior to image acquisition, cells were doubly transfected with 200 nM of SMARTpool siRNA targeting firefly luciferase (siGENOME non-targeting siRNA pool #2; D-001210-02-05; Dharmacon, Chicago, IL) or a RNA duplex targeting human μ2-adaptin [18] in OPTIMEM (Gibco, Invitrogen Corporation) using Oligofectamine (Invitrogen Corporation) according to the manufacturer's instructions. Six hours after each transfection, DMEM containing 10% FBS was added to each sample, and cells were incubated for 48 h at 37°C. The efficiency of μ2 depletion was assessed by exposing cells to 50 µg ml^−1^ Alexa 568-labeled human transferrin (Molecular Probes, Invitrogen Corporation) for 5 min. prior to addition of virus particles.

### Viral gene expression assay

Luciferase assays were performed in 96-well plates (PerkinElmer) seeded with 8,000 cells per well 16–18 h before infection. Where indicated, cells were treated with siRNA or specific chemical inhibitors. Cells were then infected with rVSV-LUC at an MOI of 0.5, and virus was removed after 1 h. At 4–5 h p.i., luminescence was measured using Steady-Glo firefly luciferase reagent (Promega) according to the manufacturer's instructions. The luminescence values were recorded on a MicroBeta TriLux scintillation counter (PerkinElmer).

### Electron microscopy

BSC-1 cells on 25 mm plastic coverslips were fixed as described previously [49]. Briefly, cells were inoculated with 20 µM cytoD in DMEM containing 10% FBS or left untreated for 10 min. at 37°C. Cells were inoculated in the presence or absence of compound with rVSV at an MOI of 5000 and incubated for an additional 15 min. Infected cells were fixed with a 37°C solution of 1% glutaraldehyde (Electron Microscopy Sciences), 0.5 mg/mL saponin (Sigma-Aldrich), and 2 mg/mL tannic acid (Mallinckrodt Inc.; St Louis, MO) in 100 mM sodium phosphate, 50 mM KCl, 5 mM MgCl_2_, pH 7.0 (Buffer A) for 30 min. at RT. Fixed cells were rinsed twice with Buffer A at pH 6.0 and treated with 1% osmiumtetroxide (OsO4)+1.5% potassiumferrocyanide (KFeCN_6_) for 30 min. Treated samples were washed in water, stained in 1% aqueous uranyl acetate for 30 min., and dehydrated in grades of alcohol (50%, 70%, 95%, 2×100%) for 5 min. each. After the dehydration, excess alcohol was blotted off on a filter paper, and the cover slips were immediately covered with a thin layer of Epon and left to polymerize at 60°C overnight. After polymerization, the plastic coverslip was peeled off and the embedded cells were glued onto a resin block and sectioned. Ultrathin sections (about 60–80 nm) were cut on a Reichert Ultracut-S microtome, picked up on to copper grids stained with uranylacetate and lead citrate. Images were acquired using a G2 Spirit Bio Twin (Fei, Hillsboro, OR) transmission electron microscope.

### Live cell imaging

Cells on 25 mm coverslips were placed into a perfusion chamber and overlayed with α-MEM lacking phenol red supplemented with 20 mM HEPES pH 7.4 and 2% FBS. The chamber was placed into a sample holder (20/20 Technology Inc.; Wilmington, NC) kept at 37°C, and the humidified air above the cells was maintained at 37°C and 5% CO_2_. To image virus internalization, cells were inoculated with Alexa Fluor 647 rVSV or rVSV eGFP-P at a MOI of ∼500 following brief centrifugation of the virus stock to eliminate aggregates. Direct visual inspection of virus particles in the stock confirmed a lack of such aggregates and presence of single, isolated viral particles (Figure S1B). Samples were sequentially illuminated at 2–8 s intervals for 50–150 ms using the previously described laser and confocal microscope configuration [1],[50].

### Image and data analysis

Virus entry and coated pit formation were analyzed using Slidebook 4.2 (Intelligent Imaging Innovations; Denver, CO). Complete virus internalization events were defined by an initial absence of adaptor or clathrin, followed by the colocalization of coat proteins with a particle and detectable displacement of the virion from its original position after coat disassembly. Only complete internalization events were included in the analyses of pit lifetime, kinetics of coat protein recruitment, and protein abundance deduced from fluorescence intensity of the tagged proteins. The following events were excluded from these analyses: (1) events in which coat proteins colocalized with virus prior to the start or were still associated at the end of a time-lapse acquisition; (2) events in which particles moved rapidly during colocalization with clathrin (endosomal virions) or failed to move after its disappearance; and (3) events in which the fluorescence of another clathrin structure collided with a virus-containing pit. Figure S3 summarizes the relative frequencies of all events analyzed in this study.

Conventional coated vesicles were identified and analyzed based on previously-defined spatial and kinetic parameters [1],[4],[51]. The rate of coated pit nucleation (Figure 7) and the efficiency of pit completion in the presence and absence of cytoD and latB (Figure 6C) were estimated using an automated image analysis application (IMAB) created with MATLAB 7 (Mathworks; Natick, MA) [4]. Images were processed and analyzed as above, and all events were verified manually. SigmaPlot 8.0 (SYSTAT; Point Richmond, CA) was used to plot data and perform statistical analyses. Movies were created using ImageJ (U. S. National Institutes of Health, Bethesda, Maryland; http://rsb.info.nih.gov/ij/).

To define the position of a particle over time, a mask encompassing the virion was manually applied to each time-lapse image and centered on the peak fluorescence intensity of the virus-associated dye. The X, Y coordinates of the mask centroid were used to calculate the particle displacement at any new position (X_n_, Y_n_) relative to the origin of virus attachment (X_0_, Y_0_) according to the equation: displacement = (X_n_−X_0_)+(Y_n_−Y_0_). The rate of particle displacement was estimated by plotting the square of the mean displacement versus time, and the diffusion coefficient (D) was calculated using the relationship: (mean displacement)^2^ = 4Dt.

The fluorescence intensity of coat-associated proteins was measured by applying a mask that encompassed the pit fluorescence. Local background was subtracted by measuring the fluorescence associated with an equal number of pixels. To obtain the fluorescent traces of individual viral events, values were scaled relative to the average maximal fluorescence of clathrin observed in pits lacking virus from the same cells. The normalized values were graphed as a weighted average of every third value, where 60% was applied to the median value and 20% to each adjacent value. Comparative plots of the internalization lifetime and protein recruitment kinetics were generated in the following manner: (1) the fluorescence intensity of each protein was averaged over time for all pits containing or lacking virus; (2) the average maximum fluorescence and total lifetime of clathrin in pits lacking virus was set to 100; (3) both parameters were normalized proportionally for virus-containing pits; (4) the protein fluorescence was plotted at 8 equally-spaced intervals+/−the standard error.

### Estimation of vesicle surface area

The surface area (SA) of spherical vesicles was estimated based on their internal radius (r), according to the equation: SA = 4πr^2^, or for a prolate spheroid with a polar radius (c) and an equatorial radius (a) using the equation: SA = 2πa^2^+2π(ac/e)sin^−1^e, where the ellipticity (e) = √1−(a^2^/c^2^).

### Monte-Carlo simulation of virus capture

MATLAB 7 (Mathworks) was used to simulate the random motion of a VSV particle on a grid of 100×100 points. Assuming a specific distance between the grid points dg (in nm), the time (t_s_) required for the particle to move between points was estimated from t_s_ = α(dg)^2^/4D, where D is the VSV diffusion coefficient (Figure 7B; 1×10^−11^ cm^2^ s^−1^), dg is the distance between two neighboring grid points, and α is a correction factor derived from calibration of the simulation. Each simulation was performed using 2.5 s step intervals. The number of coated pits in the first step was estimated as follows: (rate of pit formation)×(pit lifetime)×(100×dg)^2^. The number of new pits in each step was calculated by (rate of pit formation)×(ts)×(100×dg)^2^. Using these parameters, a VSV particle with a footprint equal to 120×120 nm^2^ was allowed to diffuse on the grid until it encountered a coated pit, and the elapsed time between the start of the simulation and particle capture was recorded for 100 particles. The average rate of pit nucleation (0.6 events per 10^8^ nm^2^ s^−1^; total 703 events) was measured in the presence of VSV for 6 cells expressing σ2-eGFP. IMAB [4] was used to identify and score all AP-2 spots that appeared during an imaging time course and exclude those that lasted fewer than 2 consecutive time points (6–8 s).

## Supporting Information

Figure S1 Visualizing individual VSV particles. (A) Virus plaque assays depicting the effect of Alexa Fluor 647 conjugation on viral titer. Purified VSV particles were labeled using the indicated concentrations of dye as described in Materials and Methods, and virions were subjected to plaque assay on Vero cells following removal of the free dye molecules. The titer of each virus stock is provided in plaque forming units per ml (pfu ml^−1^). (B) Net fluorescence intensity of VSV particles labeled with Alexa Fluor 647. Virions on a glass coverslip were imaged by confocal fluorescence microscopy, and the fluorescence intensity (arbitrary units) of each particle minus the local background was measured. The distribution of particle fluorescence intensities is displayed as a histogram plot in which the red line depicts the best fit Gaussian curve. The inset shows a representative image of virus particles (blue).
(1.17 MB TIF)

Figure S2 Parameters influencing the simulated time to VSV capture by clathrin. (A) Effect of altering the VSV diffusion coefficient (black) and the rate of coated pit nucleation (grey) on the elapsed time between onset the of virus diffusion and capture by a coated pit. The Monte-Carlo simulation was run for 100 VSV particles using the indicated alterations in each parameter while maintaining a constant virus footprint (dg) of 120×120 nm^2^ and a pit lifetime of 20 s. The pit nucleation rate was set to 0.6 events / 10^8^ nm^2^ s^−1^ when the diffusion coefficient was altered, and the latter was set to 1×10^−11^ cm^2 ^s^−1^ when the nucleation rate was changed. (B) Effect of altering the pit lifetime (black) and virus footprint size (grey) on the elapsed time between the onset of virus diffusion and capture by a coated pit. Simulations were run as for panel A.
(0.21 MB TIF)

Figure S3 Fate of all membrane-bound VSV particles analyzed in this study. (A) Fate of virions (n = 522 from 28 cells) already attached to the cell surface at the onset of image acquisition. The fate of each particle was categorized according to the nature of its association with clathrin, and a description of each outcome is displayed, along with the number of particles in each category. The box represents the total length of a representative time-lapse acquisition (ranged from 6–10 min.), and the left and right sides of the box correspond to the first (start) and last (end) image acquired, respectively. (B) Fate of virions (n = 144 from 28 cells) that attached to the cell surface during image acquisition. Particle fates are depicted as in A.
(4.94 MB TIF)

Video S1 Clathrin-dependent uptake of VSV. Time lapse depicting the internalization of single VSV virions (blue) by an individual BSC-1 cell (same as in Figure 1A) co-expressing tom-LCa (red) and σ2-eGFP (green). A portion of the bottom cell surface is shown as an overlay of the three channels, and three examples of virus internalization events are highlighted by blue circles surrounding the virions (blue). The frame rate in this and all subsequent videos was increased by 10-fold relative to real time, which is provided in the timestamp.
(2.45 MB AVI)

Video S2 AP2 and clathrin recruitment during VSV internalization. A zoomed view of the cell depicted in Video S1 highlighting three additional virus uptake events. The second circled virion corresponds to the internalization event described in Figure 1C and 1D.
(8.84 MB AVI)

Video S3 Dynamin recruitment during internalization of VSV. An overlay of the VSV (blue), tom-LCa (red), and dyn2-eGFP (green) channels is shown, and the virion of interest is circled.
(3.20 MB AVI)

Video S4 Effect of dynasore on VSV internalization. BSC-1 cells expressing tom-LCa (red) and σ2-eGFP (green) were inoculated with VSV particles (blue), and dynasore was added to 80 µM after 5 min. The video depicts two VSV particles (circled) in coated pits at the start of a time-lapse acquisition that began 12 min post-addition of dynasore. Note that neither particle is internalized.
(4.00 MB AVI)

Video S5 Recruitment of auxilin1 during VSV internalization. The movie depicts the internalization event shown in Figure 4A. The left panel is an overlay of the VSV (blue), tom-LCa (red), and eGFP-auxilin1 (green) channels, and the right panel shows only the aux1 fluorescence. The position of the virion is indicated by a circle in both panels.
(6.61 MB AVI)

Video S6 Actin dynamics during VSV internalization. The video depicts the event shown in Figure 5A. The left panel is an overlay of the VSV (blue), tom-LCa (red), and actin-eGFP (green) channels, and the right panel shows the actin fluorescence alone. The position of the virion is indicated by a circle in both panels.
(6.01 MB AVI)

Video S7 Arp3 accumulation during VSV internalization. The left panel is an overlay of the VSV (blue), tom-LCa (red), and arp3-eGFP (green) channels, and the right panel shows the arp3 fluorescence alone from the virus internalization event shown in Figure 5C. The position of the virion is indicated by a circle in both panels. Note the transient increase in arp3 fluorescence shortly before virus uptake.
(4.15 MB AVI)

Video S8 Cortactin accumulation during VSV internalization. The left panel is an overlay of the VSV (blue), tom-LCa (red), and cortactin-eGFP (green) channels, and the right panel shows the cortactin fluorescence alone from the event shown in Figure 5E. The position of the virion is indicated by a circle in both panels. Note the burst in the cortactin fluorescence shortly before virus uptake.
(8.41 MB AVI)

Video S9 Effect of cytochalasin D on VSV internalization. BSC-1 cells expressing tom-LCa (red) were treated with 20 µM cytochalasin D (cytoD) for 10 min and inoculated with VSV particles (blue). Time-lapse images were acquired starting 1 min after virus addition. The video depicts a single virion (circled) that attaches to the cell surface 15 min post-addition of cytoD and becomes trapped in a coated pit. An overlay of the VSV and LCa channels is shown.
(5.60 MB AVI)
