# Supplementary figures and images for: Vesicular Stomatitis Virus Enters Cells through Vesicles Incompletely Coated with Clathrin That Depend upon Actin for Internalization
Source: PLoS Pathog. 2009 Apr 24;5(4):e1000394. doi: 10.1371/journal.ppat.1000394 (PMC2667253; doi:10.1371/journal.ppat.1000394)

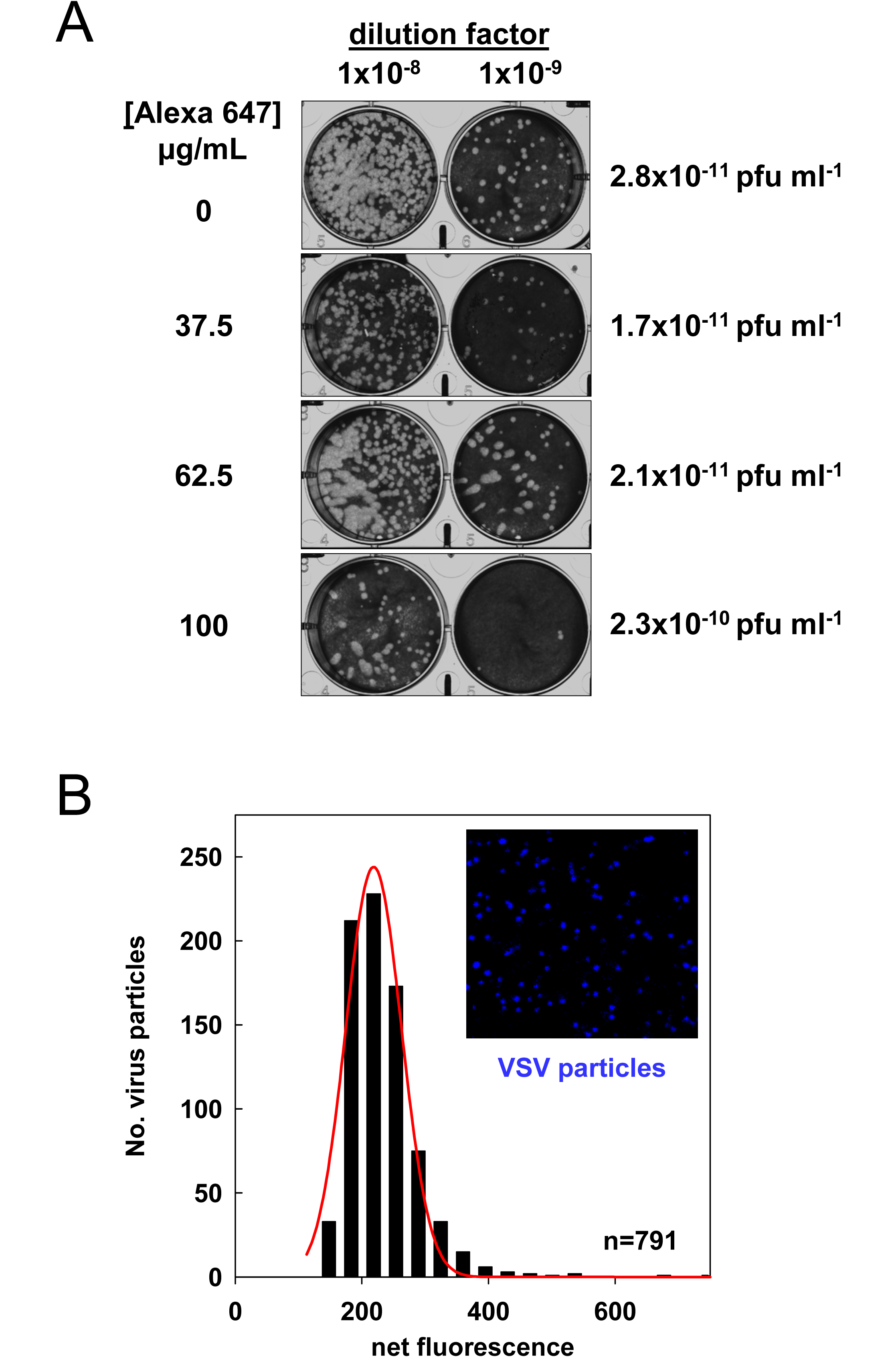

Supplement: Figure S1 — Visualizing individual VSV particles. (A) Virus plaque assays depicting the effect of Alexa Fluor 647 conjugation on viral titer. Purified VSV particles were labeled using the indicated concentrations of dye as described in Materials and Methods, and virions were subjected to plaque assay on Vero cells following removal of the free dye molecules. The titer of each virus stock is provided in plaque forming units per ml (pfu ml−1). (B) Net fluorescence intensity of VSV particles labeled with Alexa Fluor 647. Virions on a glass coverslip were imaged by confocal fluorescence microscopy, and the fluorescence intensity (arbitrary units) of each particle minus the local background was measured. The distribution of particle fluorescence intensities is displayed as a histogram plot in which the red line depicts the best fit Gaussian curve. The inset shows a representative image of virus particles (blue). (1.17 MB TIF) [file ppat.1000394.s001.tif]

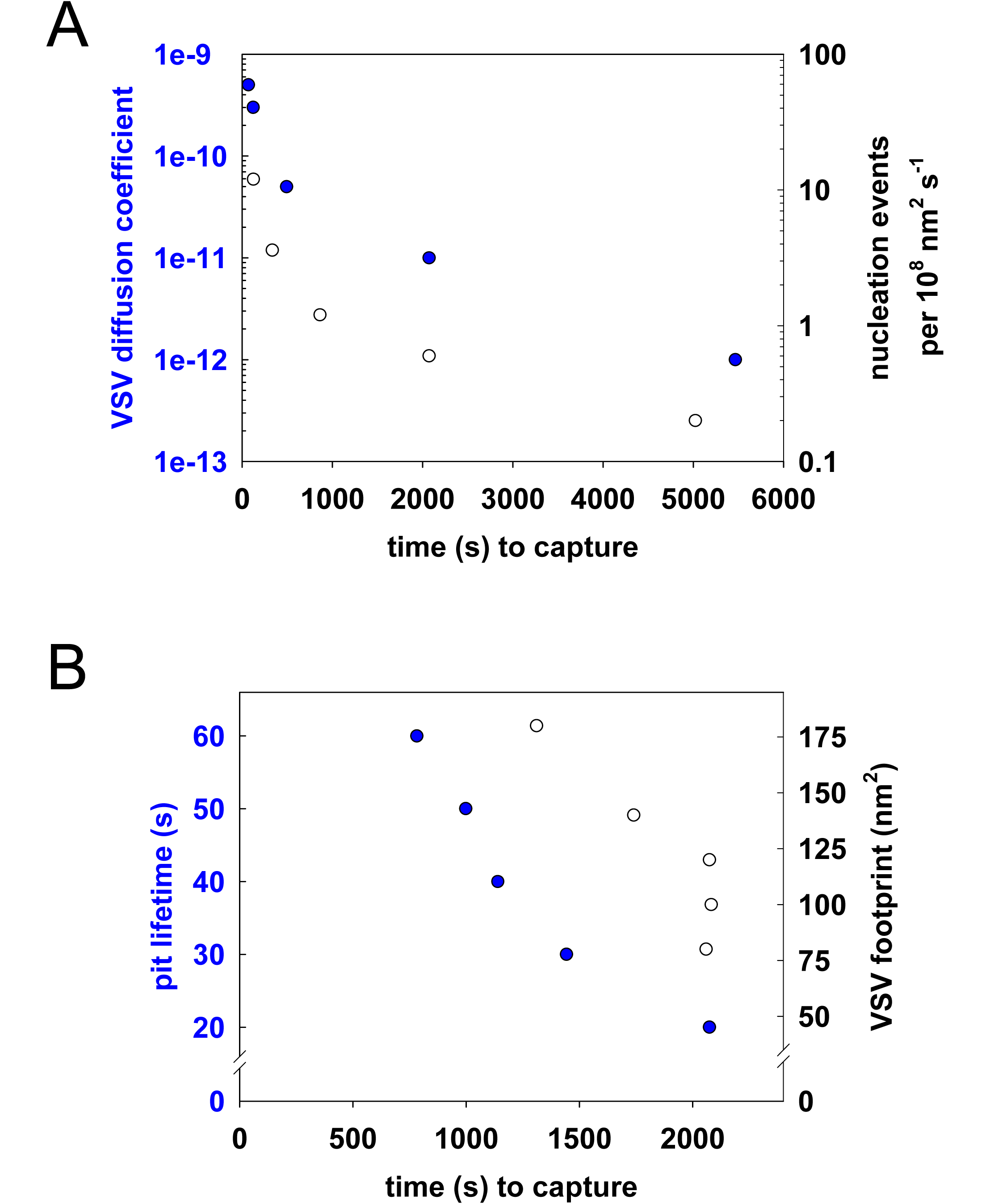

Supplement: Figure S2 — Parameters influencing the simulated time to VSV capture by clathrin. (A) Effect of altering the VSV diffusion coefficient (black) and the rate of coated pit nucleation (grey) on the elapsed time between onset the of virus diffusion and capture by a coated pit. The Monte-Carlo simulation was run for 100 VSV particles using the indicated alterations in each parameter while maintaining a constant virus footprint (dg) of 120×120 nm2 and a pit lifetime of 20 s. The pit nucleation rate was set to 0.6 events / 108 nm2 s−1 when the diffusion coefficient was altered, and the latter was set to 1×10^−11^ cm2 s−1 when the nucleation rate was changed. (B) Effect of altering the pit lifetime (black) and virus footprint size (grey) on the elapsed time between the onset of virus diffusion and capture by a coated pit. Simulations were run as for panel A. (0.21 MB TIF) [file ppat.1000394.s002.tif]

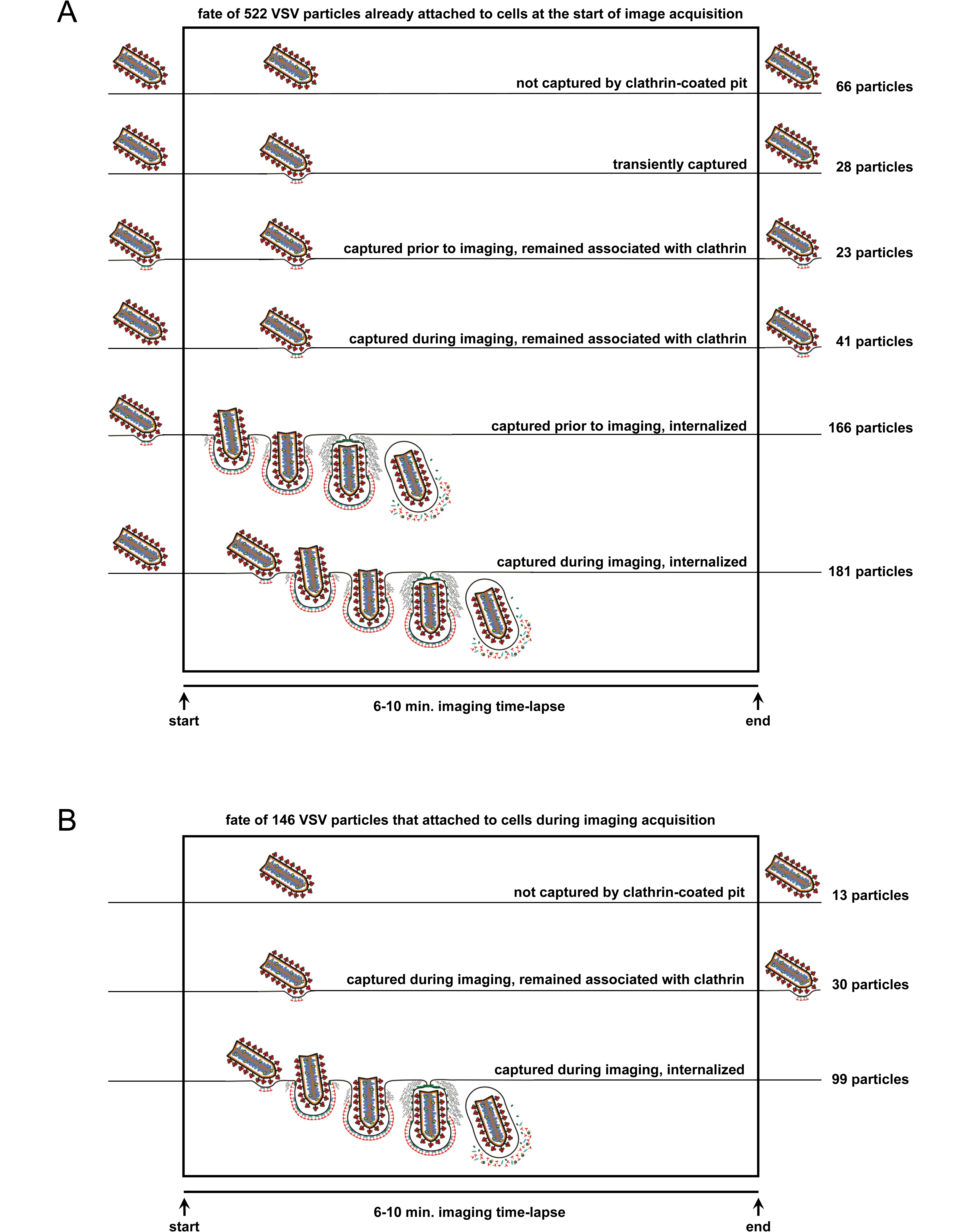

Supplement: Figure S3 — Fate of all membrane-bound VSV particles analyzed in this study. (A) Fate of virions (n = 522 from 28 cells) already attached to the cell surface at the onset of image acquisition. The fate of each particle was categorized according to the nature of its association with clathrin, and a description of each outcome is displayed, along with the number of particles in each category. The box represents the total length of a representative time-lapse acquisition (ranged from 6–10 min.), and the left and right sides of the box correspond to the first (start) and last (end) image acquired, respectively. (B) Fate of virions (n = 144 from 28 cells) that attached to the cell surface during image acquisition. Particle fates are depicted as in A. (4.94 MB TIF) [file ppat.1000394.s003.tif]
